# Separate and joint associations of chronic pain, multisite pain and mental health with sickness absence among younger employees: a register based longitudinal study

**DOI:** 10.1186/s13690-023-01115-1

**Published:** 2023-05-30

**Authors:** Pi Fagerlund, Rahman Shiri, Johanna Suur-Uski, Sara Kaartinen, Ossi Rahkonen, Tea Lallukka

**Affiliations:** 1 Department of Public Health, University of Helsinki, Tukholmankatu 8 B, P.O. Box 20, 00014 Helsinki, Finland; 2 Finnish Institute of Occupational Health, Helsinki, Finland; 3 Department of Physical and Rehabilitation Medicine, Helsinki University Hospital, Hyvinkää, Finland

**Keywords:** Chronic pain, Multisite pain, Mental health, Sickness absence, Sick leave, Employees

## Abstract

**Background:**

Pain conditions and poorer mental health are associated with work disability. However, few studies have examined the association of concurrent pain and poorer mental health with sickness absence among younger employees. We examined separate and joint associations of chronic pain, multisite pain, and mental health with total and long-term all-cause sickness absence days among younger Finnish municipal employees.

**Methods:**

The Young Helsinki Health-study data were collected in 2017 from 19–39-year-old employees of the City of Helsinki, Finland. Chronic (≥ 3 months) and multisite (≥ 2 body sites) pain and mental health (RAND-36 emotional wellbeing subscale dichotomized by median score) were self-reported (*n* = 3911). Chronic pain, multisite pain and mental health were analyzed separately and combined. Register data on total (≥ 1 workdays) and long-term (≥ 11 workdays) sickness absence days during the following year were obtained. Negative binomial regression analyses were performed with sociodemographic, socioeconomic, and health-related factors as confounders. Gender interaction and synergistic indices were examined.

**Results:**

Chronic multisite pain was associated with long-term sickness absence days (rate ratio [RR] 2.51, 95% CI 1.17–5.42). Chronic pain (RR 5.04, 95% CI 2.14–11.87) and multisite pain (RR 4.88, 95% CI 2.30–10.33) were associated with long-term sickness absence days among employees with poorer mental health. There was a synergistic interaction between gender and multisite pain for total sickness absence days (synergy index 1.80, 95% CI 1.27–2.54), with stronger associations among women.

**Conclusions:**

Chronic and multisite pain are associated with long-term sickness absence among younger employees, particularly among women and employees with concurrent poorer mental health. Consideration of this knowledge at workplaces and in healthcare could help to identify and support employees at increased risk of later sickness absence.

**Supplementary Information:**

The online version contains supplementary material available at 10.1186/s13690-023-01115-1.

**Table:** 

Text box 1. Contributions to literature
• The association of pain, concurrent mental health and sickness absence among younger employees is little studied although musculoskeletal and mental disorders dominate as reasons for long-term sickness absence
• We present findings on the associations of chronic and multisite pain and sickness absence among Finnish employees under 40 years and consider the role of concurrent mental health. We also highlight gender differences to the association between pain and sickness absence
• We suggest further intervention studies and that the pain burden among younger employees is addressed at workplaces, in healthcare and at policy making level to prevent pain related work disability

## Background

Pain conditions are common already among younger employees and may have both personal and societal consequences in terms of suffering, work disability, productivity loss, and healthcare costs [1–5]. Up to one fifth of Finnish municipal employees under 40 years report chronic pain [5]. The association between pain and poorer mental health is well established, particularly for chronic (duration ≥ 3 months) and multisite (involving ≥ 2 body sites) pain conditions [6–8].

The most common reason for long-term sickness absence (SA) among younger employees are mental disorders, followed by musculoskeletal diseases, which often present with pain as symptom [9]. The independent associations between pain and poorer mental health in relation to SA are well established, and concurrent symptoms are known to predict poorer clinical outcomes [10–13]. Nevertheless, studies on the association of concurrent pain and poorer mental health with SA have so far been conflicting and their comparability complicated by methodological heterogeneity [4, 14–18]. A population-based study conducted among six European countries found an additive association between concurrent pain and depression in relation to self-reported work loss days [16]. Further, an Australian questionnaire study identified pain and poorer mental health as predictors of self-reported SA, but no interaction of concurrent symptoms to the risk of SA was observed [4].

Most previous studies have relied on self-reported SA, but some Nordic studies have utilized register-based SA data [14, 15, 19]. Among 40–60-year-old Finnish employees, a synergistic association between concurrent chronic pain and emotional exhaustion was observed in relation to long-term SA [14]. Another Finnish study of 30–64-year-old employees found that those with concurrent pain and depressive symptoms had more long-term SA days than those with either symptom, but interaction was not assessed [19]. A Danish study focusing on female healthcare workers found no association for concurrent multisite pain and depression with long-term SA [15]. This is, to our best knowledge, the only study that has examined concurrent multisite pain and poorer mental health in relation to register based SA [15].

The role of concurrent pain and poorer mental health to the risk of SA thus needs to be clarified particularly among younger employees. This age group has the highest proportion of mental health related long-term SA, but also a significant SA burden related to musculoskeletal disorders [12]. Most long-term SA among younger Finnish employees is due to mental disorders, particularly mood- and anxiety disorders, to which pain conditions are a known comorbidity [12]. Examining concurrent pain and mental health is justified, as this provides a more detailed picture of symptomatology that may indicate a risk of later SA among employees in this age group. It is for the same reason important to understand the significance of pain characteristics, such as chronicity and number of pain sites, and concurrent mental symptoms to the association with SA.

Diagnosis-specific Finnish national register data exists for long-term SA, which is strongly linked to later disability retirement [20]. Moreover, frequent shorter SA spells are associated with long-term SA due to mental disorders among younger employees [21]. Thus, the total SA burden is also relevant. The higher SA frequency and pain prevalence among women also requires consideration [14].

Based on existing knowledge, our overall assumption is that chronic and multisite pain and concurrent poorer mental health predict less favorable SA outcomes among younger employees. To test this, we 1) examine separate and joint associations of chronic pain, multisite pain, and poorer mental health at baseline with the number of total and long-term SA days during a 1-year follow-up among younger municipal employees, 2) test interaction between chronic pain, multisite pain and poorer mental health in relation to total SA days, and 3) examine whether the associations of chronic pain, multisite pain and poorer mental health with total SA days or interaction between pain and poorer mental health in relation to total SA days differ between genders.

## Methods

### Study population

The data were derived from the Young Helsinki Health Study cohort, which follows the health and wellbeing of employees of the City of Helsinki who were 18 − 39 years at baseline in 2017 [22]. The target population were current employees of the City of Helsinki born in 1978 or later who had a regular working contract of > 50% for over four months prior to the survey. These criteria were met by 11 459 employees of whom 51% (*n* = 5898) responded. A majority (79%) of the respondents were women. Responses were obtained by web-based (58%) and mailed (29%) questionnaires. Employees who did not respond despite reminders were interviewed by phone (13%). More detailed information on the data collection is provided elsewhere [22].

**Fig. 1:**
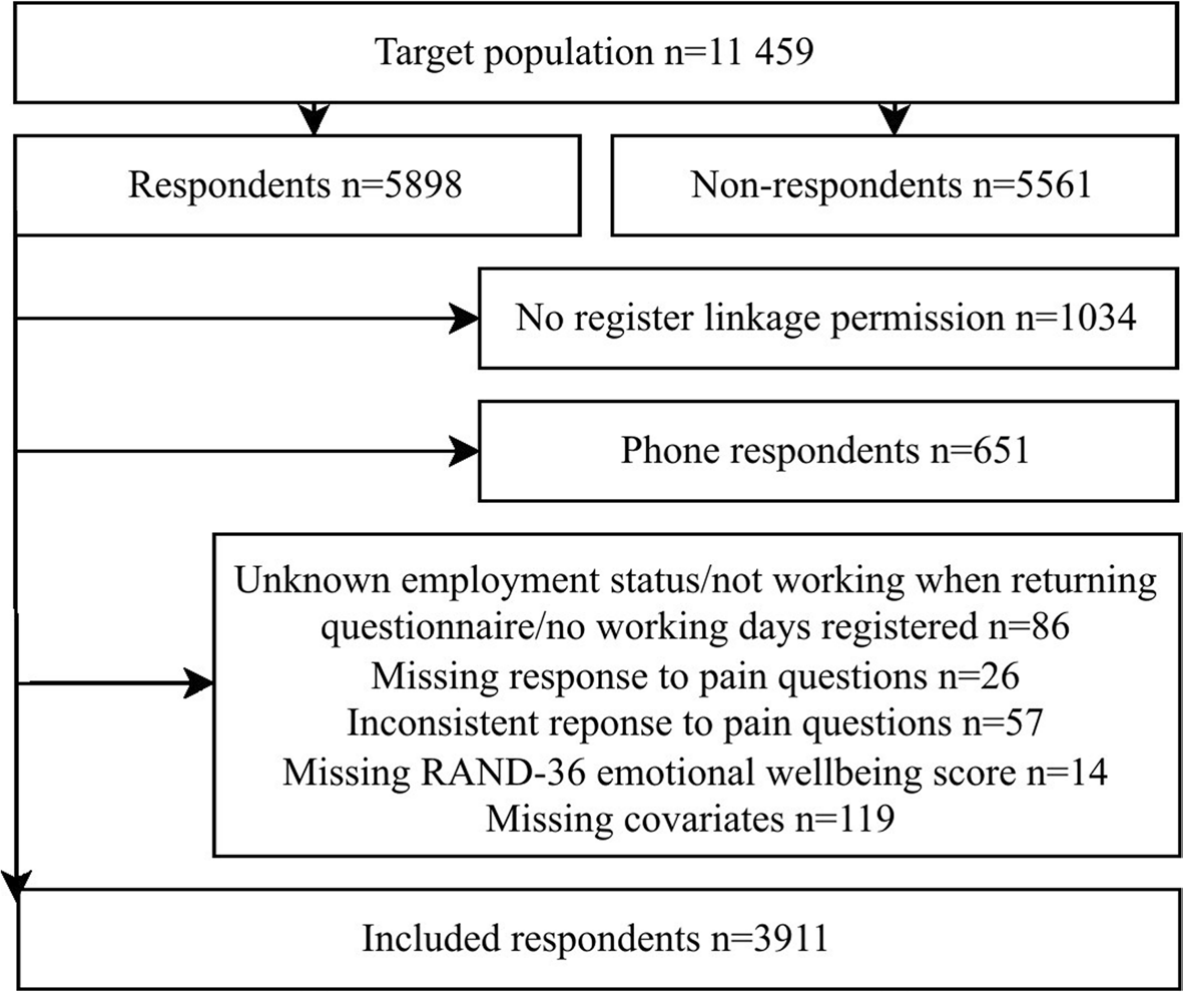
Flow chart of study participants

Inclusion criteria are presented in Fig. 1. Respondents who declined register linkage (18%) were excluded. Respondents interviewed on phone (11%) were excluded from the analyses, as all key information needed for this study was not asked during these interviews (such as the mental health measure and key covariates). Respondents who did not provide information on employment status or who had been on sickness absence for over 6 months, disability pension or rehabilitation subsidy when returning the questionnaire, and respondents whose working contract ended between submitting and returning the questionnaire were excluded (1.5%). Respondents who did not provide information on current pain chronicity and number of pain sites were excluded (0.4%). Respondents giving inconsistent responses to pain questions (e.g. reporting pain chronicity or number of pain sites although reporting no pain, which was technically possible in the mailed questionnaire) were excluded (1.0%). Respondents with missing RAND-36 emotional wellbeing score (0.2%) and missing covariates (2.0%) were excluded, except from respondents with missing information on binge drinking, who were kept as a separate category due to a high proportion of missing responses. The final analytical sample consisted of 3911 (66%) respondents.

### Pain

Current pain status was determined by the question “Are you suffering from any pains or aches right now?” (no/yes). Respondents reporting pain were further asked “When did the pain begin?” (up to 3 months ago/more than 3 months ago). Pain with a duration of ≥ 3 months was classified as chronic, otherwise as acute or subacute pain (from here on referred to as acute pain) [6]. Respondents with current pain were asked “Where do you feel the pain?” and were provided response alternatives including five specified body locations (neck and shoulders, lower back, upper extremities, lower extremities, face or head) and “elsewhere”. Each of the six response alternatives was considered as one pain site. A missing answer was interpreted as no pain at that body site. A summed score for the number of painful body sites was calculated and dichotomized as single site pain or multisite pain (≥ 2 pain sites). To examine the role of different pain characteristics to the association with SA, three pain measures were created based on chronicity (no pain/acute pain/chronic pain), number of pain sites (no pain/single site pain/multisite pain), and the combination of these (no pain/acute single site pain/acute multisite pain/chronic single site pain/chronic multisite pain).

### Mental health

Mental health was assessed by the RAND-36 emotional well-being subscale consisting of five questions reflecting mental well-being during the last four weeks (“Have you been a very nervous person?”, “Have you felt so down in the dumps that nothing could cheer you up?”, “Have you felt calm and peaceful?”, “Have you felt downhearted and blue?”, and “Have you been a happy person?”) [23, 24]. Symptoms were reported on a six-level scale ranging from “none of the time” to “all of the time”. A summed score (range 0–100 points) was calculated and dichotomized with the median value (76.0 points) as cut-off [23]. Respondents with a score below or equal to the median were defined as having poorer mental health, respondents with a score above the median as having better mental health. This cut off was chosen to enable a dichotomized stratification of pain variables by mental health. This cut off maintained sufficient statistical power for stratified analyses, but also allowed milder and earlier mental symptoms to be captured. However, identifying a subgroup with clinically significant symptoms was not possible with this approach, a limitation which we acknowledge.

### Sickness absence

In Finland, all individuals aged 16–67 years are entitled to sickness allowance. For the employee, SA days of up to 10 working days (calendar days excluding mid-week holidays and Sundays) is generally compensated by the employer and SA days exceeding 10 working days are compensated by the Social Insurance Institution of Finland (SII) for up to 300 working days [25]. A medical certificate is required for longer SA spells covered by the employer (usually after three working days) and for sickness allowance covered by the SII.

Data on SA were collected prospectively during one year from the day after the questionnaire was returned. Register data on SA days were obtained from the City of Helsinki’s personnel register and from the SII register. Longer SA spells than 10 working days are covered by the public social security system and indicate a particularly high risk of disability pension, why separate analyses of these were considered justified [20]. Two variables measuring SA were thus included:1) all SA days during the 1-year follow-up (including short self-certified spells, longer medically certified spells covered by the employer, and spells exceeding 10 working days partially covered by the Social Insurance Institution) and 2) SA-days of long-term SA spells exceeding 10 working days.

### Covariates

Sociodemographic and socioeconomic covariates included were gender, age, marital status, and education level. Health-related covariates included binge drinking, nicotine use, leisure-time physical activity and body mass index (BMI) [26]. The inclusion of covariates was based on previously documented relationship with pain and SA. Women report more pain and have more SA [11]. Older employees have more long-term SA and show a higher prevalence of pain [26, 27]. Living single is associated with sickness absence [28, 29]. Lower education level is associated with pain and SA [26, 30]. Binge drinking is associated with more SA and there is a link between alcohol consumption and chronic pain [26, 31, 32]. Smoking, low physical activity, and higher BMI are all associated with chronic pain and SA [26, 30, 33].

Gender was reported and classified as man or woman. Age was dichotomized as < 30 years and 30–39 years. Marital status was classified as married or cohabiting, and other (unmarried, divorced, or widowed). The respondent’s highest education was classified as high (master’s degree or higher), intermediate (bachelor’s degree), or low (upper secondary school or lower). Respondents using ≥ 6 alcohol servings per occasion weekly or more frequently were classified as binge drinkers. Respondents who used cigarettes, snuff, or electronic cigarettes daily or sporadically were classified as nicotine users. Leisure-time physical activity was measured in metabolic equivalent of task hours per week (MET-hours per week) based on self-reported weekly leisure-time activities equivalent to walking, brisk walking, jogging, and brisk running. Low activity was defined as < 14 MET-hours per week, intermediate activity as ≥ 14 MET-hours per week including only activity equivalent to walking, and high activity as ≥ 14 MET-hours per week including activity equivalent to jogging or running [34]. BMI was calculated based on self-reported weight and height and classified as < 25 kg/m^2^, 25–29.9 kg/m^2^ (overweight) and ≥ 30 kg/m^2^ (obesity) [35].

### Statistical methods

The total and long-term SA day counts were calculated as the number of total SA days, and days of a long-term SA spell exceeding 10 days of absence (SA days reimbursed by the SII), respectively, occurring within 12 months divided by the number of person-years. The associations of pain and poorer mental health with total or long-term SA days were assessed by negative binomial regression analysis using the log link function. The City of Helsinki’s personnel register contained data on SA for the duration of the working contract whereas data from the Social Insurance Institution were collected for one year irrespective of employer at the time of SA. During the 1-year follow-up, 14% of the work contracts ended. To control for differences in follow-up time, the natural logarithm of the number of days of employment during the follow up year was used as an offset variable for the analyses of total SA days. Sensitivity analyses to assess the effect of excluding the 14% of employees whose working contract ended during the follow-up, were performed. Long-term SA data were complete for one year and did not require adjustment. All models were adjusted for gender and age. Model 2 was further adjusted for marital status, education, binge drinking, nicotine use, physical activity, and BMI. To avoid residual confounding, age and BMI were adjusted for as continuous variables in the regression analyses.

We further explored whether there is a synergistic interaction between pain and poorer mental health for the risk of SA. A synergistic interaction refers to a joint effect of two factors that is greater than the sum of their individual effects [36, 37]. First, stratified analyses were performed based on pain chronicity (no pain/acute pain/chronic pain), number of pain sites (no pain/single site/multisite pain), and mental health (better mental health/poorer mental health). The synergistic interaction between pain and mental health was assessed by the synergy index (SI) which describes the rate ratio in the double-exposed group (concurrent pain and poorer mental health) in relation to the added rate ratios of the single-exposed groups. The SI was calculated using the Excel-formula provided by Andersson et al. by the equation [RR (AB) – 1]/[(RR(Ab) – 1 + (RR (aB) – 1)], where A and B denote the two predictors (pain and poorer mental health) and a and b denote the absence of these predictors (no pain and better mental health) [38, 39]. A SI > 1 suggests a positive synergistic interaction, meaning that the joint effects of pain and poorer mental health to sickness absence are more than additive [40–42]. Furthermore, gender stratified analyses were conducted, and synergy indices were estimated for the interaction between pain, gender, and SA [38, 39].

Missing values for covariates ranged from 0.1% to 1.2%, but missing data for binge drinking were 3.7%. Crude complete case analyses did not differ significantly from crude imputed data analyses. However, respondents with missing information on binge drinking had a higher number of SA days. To reduce selection bias, the respondents with missing data on binge drinking were classified as a separate category, while respondents with missing data on other covariates (*n* = 119) were excluded from the analyses. All statistical analyses were performed using IBM SPSS version 27. Graphs visualizing the study design are presented in Additional figure 1.

### Ethical considerations

The Young Helsinki Health Study plan was approved by the City of Helsinki and obtained a positive statement by the ethical board of the Faculty of Medicine at the University of Helsinki. All included participants gave their informed consent to register linkage.

## Results

### Descriptive results

The mean number of total SA days was 12.1 days per person-year and the mean number of long-term SA days was 4.4 days (Table 1). The number of SA days was higher among women, those who were younger than 30 years, unmarried, had low education, did not report alcohol use, used nicotine, or were overweight or obese. Mental disorders accounted for nearly half of the long-term SA days (Additional table 3).

**Table 1:** Descriptive characteristics of the study population by total and long-term sickness absence days during 1- year follow-up. Percentages and mean sickness absence days per person years

		**Total SA days**	**Long-term SA days**
	%	mean (95% CI)	mean (95% CI)
**Gender**			
Women (*n* = 3141)	80.3	12.8 (11.7–13.9)	4.5 (3.8–5.3)
Men (*n* = 770)	19.7	9.3 (8.0–10.7)	3.9 (2.5–5.2)
All (*n* = 3911)	100.0	12.1 (11.2–13.0)	4.4 (3.8–5.0)
**Age**			
< 30 years	31.3	15.2 (13.0–17.4)	5.7 (4.3–7.0)
≥ 30 years	68.7	10.7 (9.8–11.6)	3.8 (3.1–4.5)
**Marital status**			
Married or cohabiting	67.2	11.0 (10.0–12.1)	3.8 (3.1–4.5)
Unmarried	32.8	14.3 (12.5–16.2)	5.6 (4.3–7.0)
**Education level**			
High	29.8	7.8 (6.5–9.1)	2.5 (1.7–3.4)
Intermediate	37.8	12.1 (10.5–13.8)	4.5 (3.5–5.6)
Low	32.4	16.0 (14.3–17.7)	6.0 (4.6–7.3)
**Binge drinking**			
Rarely	90.0	13.3 (8.8–17.8)	4.4 (3.7–5.0)
Weekly or more	6.4	11.9 (11.0–12.7)	4.4 (1.9–6.9)
Missing response	3.7	16.7 (6.3–27.0)	5.2 (1.3–9.0)
**Nicotine use**			
Never or cessated	72.8	11.0 (10.0–12.0)	3.9 (3.2–4.6)
Daily or sporadical	27.2	15.1 (13.1–17.1)	5.6 (4.2–7.1)
**Physical activity**			
High	61.8	10.7 (9.7–11.8)	3.8 (3.0–4.5)
Intermediate	28.7	14.5 (12.3–16.8)	5.7 (4.3–7.2)
Low	9.5	13.7 (11.5–15.9)	4.5 (2.9–6.1)
**Body mass index (kg/m**^**2**^**)**			
< 25	59.1	10.8 (9.7–12.0)	3.4 (2.7–4.1)
25–29.9	26.4	11.9 (10.4–13.4)	5.2 (3.8–6.7)
≥ 30	14.5	17.7 (14.6–20.9)	7.1 (5.0–9.1)

*CI* Confidence interval

Current pain was reported by 43%, chronic pain by 20%, multisite pain by 28%, and chronic multisite pain by 13% of the respondents (Table 2). Employees with pain, particularly multisite pain, had poorer mental health to a higher degree than employees without pain. Women reported pain and had poorer mental health more frequently than men.

**Table 2:** Pain and mental health by gender and by total and long-term sickness absence days during 1- year follow-up. Percentages and mean sickness absence days per person years

	**Gender**			**Sickness absence days**	
	**Total**	**Women**	**Men**	**Total**	**Long-term**
	%	%	%	mean (95% CI)	mean (95% CI)
**Pain chronicity**					
No pain	57.5	55.8	64.4	9.5 (8.5–10.5)	3.2 (2.4–4.0)
Acute	22.7	23.8	17.9	13.9 (12.5–15.3)	5.6 (4.1–7.1)
Chronic	19.8	20.4	17.7	17.7 (14.5–20.9)	6.5 (4.9–8.1)
**Pain sites**					
No pain	57.5	55.8	64.4	9.5 (8.5–10.5)	3.2 (2.4–4.0)
Single site	14.4	14.5	13.9	12.4 (10–14.8)	3.9 (2.5–5.3)
Multisite	28.1	29.6	21.7	17.3 (15.1–19.6)	7.1 (5.6–8.6)
**Mental health**					
Better	47.1	46.7	48.8	8.7 (7.9–9.5)	2.3 (1.8–2.8)
Poorer	52.9	53.3	51.2	15.1 (13.6–16.7)	6.3 (5.2–7.4)
**Pain chronicity by pain locations**					
No pain	57.5	55.8	64.4	9.5 (8.5–10.5)	3.2 (2.4–4.0)
Acute single site	8.0	8.0	7.9	11.0 (8.9–13.1)	4.3 (2.3–6.3)
Acute multisite	14.7	15.8	10.0	15.4 (13.6–17.3)	6.3 (4.3–8.4)
Chronic single site	6.4	6.5	6.0	14.1 (9.5–18.8)	3.4 (1.5–5.3)
Chronic multisite	13.4	13.8	11.7	19.4 (15.1–23.7)	8.0 (5.7–10.2)
**Pain chronicity by mental health**					
No pain, better mental health	30.3	29.1	35.5	7.0 (6.3–7.7)	1.7 (1.1–2.3)
No pain, poorer mental health	27.2	26.7	29.0	12.3 (10.4–14.2)	4.9 (3.4–6.4)
Acute, better mental health	9.7	10.2	7.7	11.9 (10.1–13.8)	3.8 (2.4–5.1)
Acute, poorer mental health	13.0	13.6	10.3	15.3 (13.3–17.4)	7.0 (4.6–9.4)
Chronic, better mental health	7.1	7.4	5.7	11.7 (8.5–15.0)	2.9 (1.2–4.6)
Chronic, poorer mental health	12.8	13.0	11.9	21.0 (16.3–25.7)	8.5 (6.1–10.8)
**Pain sites by mental distress**					
No pain, better mental health	30.3	29.1	35.5	7.0 (6.3–7.7)	1.7 (1.1–2.3)
No pain, poorer mental health	27.2	26.7	29.0	12.3 (10.4–14.2)	4.9 (3.4–6.4)
Single site, better mental health	6.8	6.9	6.5	9.2 (7.0–11.5)	2.2 (0.6–3.7)
Single site, poorer mental health	7.6	7.6	7.4	15.2 (11.3–19.2)	5.4 (3.2–7.7)
Multisite, better mental health	9.9	10.7	6.9	13.6 (11.2–16.1)	4.3 (2.8–5.7)
Multisite, poorer mental health	18.1	18.9	14.8	19.4 (16.2–22.6)	8.7 (6.5–10.9)

*CI* Confidence interval

### Associations of pain chronicity, pain sites, and mental health with sickness absence

Acute and chronic pain were associated with the total number of SA days (Table 3), however, only chronic pain was associated with long-term SA days after adjusting for gender and age. Further adjustment for sociodemographic, socioeconomic, and health-related factors slightly attenuated the associations. Chronic pain remained associated with long-term SA after full adjustment for covariates (rate ratio [RR] 1.96, 95% CI 1.01–3.79).

**Table 3:** Associations of pain chronicity, pain sites, mental health, and their interactions with total and long-term sickness absence days per person years during 1- year follow-up

	**Total sickness absence**			**Long-term sickness absence**	
	**Model 1**	**Model 2**	**Synergy index**	**Model 1**	**Model 2**
	RR (95% CI)	RR (95% CI)		RR (95% CI)	RR (95% CI)
**Pain chronicity**					
No pain	1.00	1.00		1.00	1.00
Acute	1.45 (1.28–1.63)	1.36 (1.21–1.53)		1.71 (0.93–3.16)	1.70 (0.92–3.15)
Chronic	1.87 (1.64–2.12)	1.66 (1.47–1.89)		2.19 (1.13–4.21)	1.96 (1.01–3.79)
**Pain sites**					
No pain	1.00	1.00		1.00	1.00
Single site	1.32 (1.14–1.52)	1.28 (1.11–1.47)		1.19 (0.57–2.46)	1.14 (0.54–2.40)
Multisite	1.81 (1.62–2.02)	1.62 (1.44–1.81)		2.34 (1.31–4.16)	2.19 (1.22–3.90)
**Mental health**					
Better	1.00	1.00		1.00	1.00
Poorer	1.70 (1.54–1.87)	1.63 (1.48–1.8)		2.81 (1.71–4.60)	2.73 (1.65–4.54)
**Pain chronicity by pain sites**					
No pain	1.00	1.00		1.00	1.00
Acute single site	1.17 (0.97–1.40)	1.16 (0.97–1.39)		1.26 (0.50–3.21)	1.33 (0.51–3.43)
Acute multisite	1.60 (1.39–1.84)	1.47 (1.28–1.69)		1.97 (0.95–4.05)	1.90 (0.92–3.93)
Chronic single site	1.51 (1.23–1.84)	1.43 (1.17–1.75)		1.09 (0.39–3.04)	0.91 (0.32–2.59)
Chronic multisite	2.05 (1.77–2.37)	1.78 (1.54–2.06)	0.95 (0.63–1.42)	2.76 (1.29–5.93)	2.51 (1.17–5.42)
**Pain chronicity by mental health**					
No pain, better mental health	1.00	1.00		1.00	1.00
No pain, poorer mental health	1.69 (1.49–1.92)	1.66 (1.47–1.89)		2.95 (1.55–5.63)	2.73 (1.39–5.37)
Acute, better mental health	1.62 (1.36–1.93)	1.55 (1.31–1.85)		2.17 (0.88–5.35)	2.12 (0.86–5.24)
Acute, poorer mental health	2.14 (1.82–2.5)	1.94 (1.66–2.27)		4.07 (1.81–9.14)	3.65 (1.59–8.35)
Chronic, better mental health	1.69 (1.38–2.07)	1.50 (1.23–1.83)		1.83 (0.65–5.13)	1.20 (0.40–3.59)
Chronic, poorer mental health	2.90 (2.47–3.41)	2.58 (2.20–3.02)	1.38 (0.99–1.91)	5.53 (2.42–12.63)	5.04 (2.14–11.87)
**Pain sites by mental health**					
No pain, better mental health	1.00	1.00		1.00	1.00
No pain, poorer mental health	1.69 (1.49–1.92)	1.66 (1.46–1.88)		2.94 (1.54–5.60)	2.72 (1.38–5.34)
Single site, better mental health	1.27 (1.04–1.56)	1.22 (1.00–1.49)		1.20 (0.43–3.39)	1.04 (0.36–2.97)
Single site, poorer mental health	2.17 (1.78–2.63)	2.11 (1.74–2.56)		3.27 (1.21–8.87)	3.17 (1.11–9.03)
Multisite, better mental health	1.91 (1.61–2.28)	1.75 (1.47–2.08)		2.61 (1.07–6.36)	2.26 (0.91–5.61)
Multisite, poorer mental health	2.66 (2.31–3.07)	2.31 (2.00–2.66)	1.03 (0.78–1.36)	5.48 (2.64–11.37)	4.88 (2.3–10.33)

Model 1 adjusted for gender and age (continuous)

Model 2 adjusted for age (continuous), gender, marital status, education level, binge drinking, nicotine use, physical activity and BMI (continuous)

*RR* Risk ratio, *CI* Confidence interval

When pain chronicity was stratified by mental health, both acute and chronic pain remained associated with total SA irrespective of mental health status after full adjustment for covariates, while acute (RR 3.65, 95% CI 1.59–8.35) and chronic pain (RR 5.04, 95% CI 2.14–11.87) were associated with long-term SA only among participants with poorer mental health.

Single site and multisite pain were associated with total SA after adjusting for gender and age (Table 3) but only multisite pain was associated with long-term SA. Further adjustment for sociodemographic, socioeconomic, and health related factors slightly attenuated the associations. Multisite pain remained associated with long-term SA after full adjustment for covariates (RR 2.19, 95% CI 1.22–3.90).

When number of pain sites was stratified by mental health, both single site and multisite pain remained associated with total SA irrespective of mental health after full adjustment for covariates but single site (RR 3.17, 95% CI 1.11–9.03) and multisite (RR 4.88, 95% CI 2.3–10.33) pain were associated with long-term SA only in participants with poorer mental health.

Acute single site pain was not associated with SA after adjusting for confounders. However, both acute (RR 1.47, 95% CI 1.28–1.69) and chronic (RR 1.78, 95% CI 1.54–2.06) multisite pain and chronic single site pain (RR 1.43, 95% CI 1.17–1.75) were associated with total SA. Only chronic multisite pain was associated with long-term SA (RR 2.76, 95% CI 1.17–5.42).

No significant synergistic interaction was found between pain chronicity and pain sites, or between pain and poorer mental health in relation to total SA. However, the synergy index for the interaction between pain and poorer mental health was above 1, indicating a possible synergy (Table 3).

Gender stratified analyses revealed higher pain prevalence and SA rates among women (Tables 1 and 2). Gender interaction analyses showed significantly stronger associations between pain and SA among women (Additional table 1). There was a synergistic interaction between multisite pain and female gender in relation to total SA (SI 1.80, 95% CI 1.27–2.54), with more total SA days among women with multisite pain than among men with multisite pain.

### Sensitivity analyses

As 14% of the working contracts ended during the follow up period, these employees had a shorter follow up time regarding total SA, but not for long-term SA, which was based on national register data and thus, was independent of the employer. After excluding these employees from the regression analyses, similar results were obtained with maintained associations between pain and poorer mental with total SA, as well as, with long-term SA. Thus, those who ended their working contract during the follow up did not disproportionally contribute to the total SA burden in this population. The reason for the ending of the working contract was not known based on this data.

## Discussion

### Main findings

We examined separate and joint associations of chronic pain, multisite pain, and poorer mental health with total and long-term SA days among younger Finnish municipal employees. Pain at baseline was associated with SA days during the following year. Chronic and multisite pain were associated with long-term SA days among those with concurrent poorer mental health. No significant synergistic interaction between pain and poorer mental health in relation to total SA was observed. The associations between pain and total SA were stronger among women and there was a synergistic interaction between gender and multisite pain in relation to total SA.

### Previous studies

A main aspect in which this study adds to existing literature is to confirm the association between pain and SA also among Finnish employees under 40 years. The prevalence of chronic pain in our cohort was slightly lower compared to among older municipal employees [5, 14]. The findings further confirm the significance of pain chronicity and number of pain sites to the risk of SA in this younger age group, and the central role of concurrent poorer mental health to the risk of long-term SA.

In our study population, chronic pain was clearly associated with long-term SA, but no association between acute pain and long-term SA days was observed. However, acute pain was associated with the total number of SA days, which include shorter SA spells. This finding was expected, as acute self-limiting pain conditions may cause temporary work disability but less likely lead to long-term SA. Yet, in aim to prevent pain related long-term SA, it is vital to prevent pain chronification through appropriate management of acute pain conditions and potential underlying pathology.

Most employees reporting chronic pain had multisite pain and we observed a clear association between multisite pain and SA, particularly long-term SA, among employees with poorer mental health. The only previous study we identified using a similar approach and register-based SA data, shows partly contrasting results [15]. This study among women working in the Danish healthcare sector, found separate associations of multisite pain and depressive symptoms with long-term SA (> 30 days), but no association with long-term SA for concurrent symptoms. The authors speculated that high work demands in the health care sector may result in selection of the most robust individuals who maintain their work ability despite symptoms. This possible selection mechanism could also be present in our study population. Methodological differences such as definition on long-term SA, measures of mental health, and the focus on health-care workers of all ages in the Danish study, could have contributed to different results in these two studies.

Concurrent pain and poorer mental health have been studied also in relation to other indicators of work disability than SA, and the results are in line with ours. In a prospective study of 50–61-year-old employees from the U.S., the association of concurrent severe pain and depressive symptoms with self-reported work-limiting health problems was examined during a six-year follow-up [17]. Reporting comorbid severe pain and depressive symptoms were associated with poorer outcome compared to reporting only depressive symptoms. Another Finnish study covering 30–64-year-old employees found a synergistic association between concurrent musculoskeletal pain and depressive symptoms with poor self-rated physical work ability and thoughts on early retirement [18].

Our separate focus on younger employees was considered justified as a different disease spectrum contributes to work disability among younger employees [12]. Moreover, the consequences of long-term work disability may be even more far-reaching for the life trajectory if it occurs at a younger age. A prospective study of 20–66-year-old Swedish twins showed that chronic multisite pain or a common mental disorder increased the risk of long-term SA, disability pension, and mortality among individuals with a history of SA [43]. The effect of concurrent pain and mental health were not examined, but the findings elucidate the importance of early recognition of pain and poorer mental health to prevent further work disability.

We found clear gender differences in the prevalence of pain and SA, as in previous population-based and occupational studies [10, 11]. Women reported more chronic and multisite pain than men, as in previous studies [14, 26, 44]. We further observed a synergistic interaction of multisite pain and female gender with sickness absence. The underlying mechanisms for gender differences in pain are not completely unraveled, but various psychosocial, hormonal, and genetic factors have been suggested to contribute to gender differences in both the experience and prevalence of pain [26]. How these mechanisms extend to reflect on work ability is complex and may involve gender differences in occupations and physical work demands. One previous Finnish study has indicated that women with multisite musculoskeletal pain to a higher degree experience good work ability compared to men [45]. However, this does not align with our finding of a synergistic interaction between multisite pain and female gender with SA, why further research to clarify these gender differences and potential work-related confounding is warranted.

Mental disorders accounted for most long-term SA in our cohort, as on Finnish national level [12]. Long-term SA due to mental disorders were over-represented among employees reporting pain, particularly multisite pain. Diagnosis-specific long-term SA data from the SII contain one primary diagnosis entitling to sickness allowance, while the clinical symptomatology often is more complex. Given the clear link between chronic and multisite pain with mental and musculoskeletal disorders, and considering the association with long-term SA, the role of pain in work disability is likely to be considerable, although not clearly visualized in SA statistics [13].

Demographic changes with an ageing population and a weakening economic dependency ratio highlight the importance of supporting the work ability of the younger employees [46]. As the current study shows, a significant proportion of younger municipal employees live and work with pain. Sociodemographic, socioeconomic, and health-related factors explained only part of the association between pain, poorer mental health, and SA. Physical and psychosocial strain at work have been linked to both pain and SA [47–49]. Occupational class differences in the association between pain and SA have been documented, and work-related factors likely contribute to the causal pathway between pain, mental health, and SA [50]. To avoid overadjustment bias, we did not adjust the analyses for work-related factors.

Although pain is associated with SA, a large proportion of affected employees maintain good work ability [45, 51]. Non-strenuous physical work, high supervisor support and physical activity are shown to protect against work disability [45]. However, high levels of fear-avoidance behavior have been associated with a higher rate of SA among employees with pain [52, 53]. Pain management, workplace relationships, and workplace adjustments are known to be central for maintaining work ability after chronic pain related SA [54]. Thus, we suggest further intervention studies to examine whether screening for pain conditions at workplaces and in occupational healthcare, in combination with low-threshold preventive, supportive, and therapeutic services, could reduce pain SA in the younger working population.

### Methodological considerations

Pain and symptoms of poorer mental health are subjective and therefore suitable for self-report, which captures all symptoms and not only those that have led to seeking healthcare. Mental health was not assessed by diagnostic tools in our questionnaire. However, the RAND-36 emotional well-being captures symptoms of affective and anxiety disorders [23, 55]. The questionnaire data were based on a large occupational cohort and covered municipal employees from various occupational sectors. We used reliable and prospective register data on total and long-term SA by combining data from the employer and the SII, in contrast with previous studies that mainly have focused on self-reported SA or only long-term SA [15, 18, 19]. Separating long-term SA from total SA enables distinguishing work absenteeism due to medically verified illness which is strongly linked to future disability retirement [20]. As part of the total SA data, we considered short-term SA spells, which are common, have financial implications, and confer a higher risk of long-term SA [21].

Nevertheless, this study had limitations. The number of men was small but reflects the gender proportion in the Finnish municipal sector [56]. The data were broadly representative of the target population. However, the excluded phone-interviewed employees were more likely to be of lower socioeconomic position, contributing to the results being conservative [22]. Accordingly, separate analyses including phone respondents revealed slightly stronger associations between pain and SA (Additional Table 2). Our data did not include private sector employees. However, it is unlikely that pain and poorer mental health only affect SA among public sector employees. Indeed, studies with nationally representative data have reported similar associations between pain, poorer mental health, and work disability, albeit not focusing specifically on younger employees [10, 57]. Non-respondents were predominately manual workers, who are known to have higher rates of pain and subjective pain related disability [5, 22]. The study was conducted in the Helsinki Capital region, where the morbidity is lower than the national level [58]. Finally, self-reported covariates may be affected by reporting bias.

## Conclusions

Chronic and multisite pain are associated with sickness absence among younger employees. The associations are generally stronger among women and particularly among those with poorer mental health. Considering this knowledge at workplaces and in healthcare could help to identify and provide support to employees at increased risk of later sickness absence.

## Supplementary Information

**Additional file 1: Additional figure 1.** Model of the study design. A) The association between pain characteristics and sickness absence, the interaction of concurrent mental health and the considered confounders. B) The age-adjusted association between pain characteristics and mental health with sickness absence and the interaction of gender. BMI=body mass index.

**Additional file 2:** **Additional table 1.** Gender stratified age-adjusted associations of pain chronicity, pain sites, and mental health with total sickness absence days per person years during 1- year follow-up. Synergy indices for gender interaction.

**Additional file 3:** **Additional table 2.** Association of pain chronicity, pain sites, and their interactions with total and long-term sickness absence days per person years during 1- year follow-up, including phone respondents.

**Additional file 4:** **Additional table 3.** Pain status by ICD-10 diagnosis specific long-term sickness absence days during 1- year follow-up.

## Data Availability

Data are available upon reasonable request. The Helsinki Health Study survey data cannot be made publicly available due to strict data protection laws and regulations. The data can only be used for scientific research and to the research group’s cooperation partners with a reasonable request and study plan. More information on the availability of the survey data can be inquired from the Helsinki Health Study research group (kttl-hhs@helsinki.fi). Register data cannot be shared.

## Abbreviations

SA: Sickness absence
SII: Social insurance institution
CI: Confidence interval
RR: Rate ratio
BMI: Body mass index
MET: Metabolic equivalent of task
