# Supplementary figures and images for: Separate and joint associations of chronic pain, multisite pain and mental health with sickness absence among younger employees: a register based longitudinal study
Source: Arch Public Health. 2023 May 30;81:97. doi: 10.1186/s13690-023-01115-1 (PMC10228037; doi:10.1186/s13690-023-01115-1)

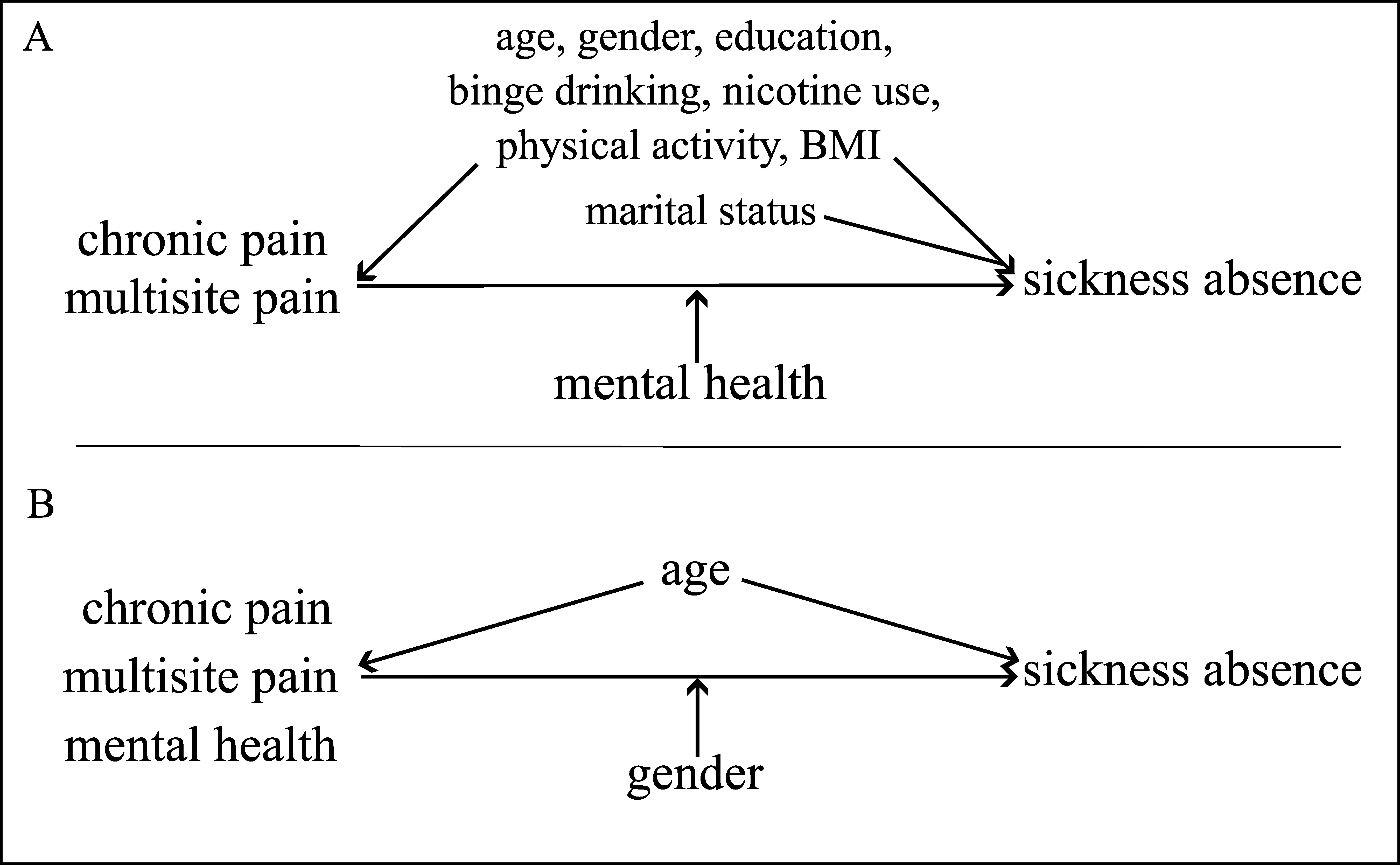

Supplement: Supplementary file 1 — Additional file 1: Additional figure 1. Model of the study design. A) The association between pain characteristics and sickness absence, the interaction of concurrent mental health and the considered confounders. B) The age-adjusted association between pain characteristics and mental health with sickness absence and the interaction of gender. BMI=body mass index. [file 13690_2023_1115_MOESM1_ESM.jpg]
